# Supplementary material for: Pleiotropic requirements for human TDP-43 in the regulation of cell and organelle homeostasis
Source: Life Sci Alliance. 2019 Sep 16;2(5):e201900358. doi: 10.26508/lsa.201900358 (PMC6749094; doi:10.26508/lsa.201900358)
Supplement: Supplementary file 3 [file LSA-2019-00358_TableS3.doc]

**Supplemental Table 3:** Summary of oligonucleotide primers used in this study.

| **Gene Target** | **Use** | **Sense sequence** | | **Antisense sequence** | |
| --- | --- | --- | --- | --- | --- |
| **TDP43** | CRISPR KO (gRNA1) | | 5'caccgacatccgatttaatagtgtt-3’ | | 5’-aaacaacactattaaatcggatgt-3’ |
| **TDP43** | CRISPR KO  (gRNA2) | | 5’-caccgcccatggaaaacaaccgaac-3’ | | 5’-aaacgttcggttgttttccatgggcggtg-3’ |
| **TDP43** | Genomic DNA amplification | | 5’-tgcaccagaaaagcacctca-3’ | | 5’-tcgaagtttacctgcaccat-3’ |
| **TDP43** | Cloning into pLVX by Gibson assembly | | 5’-tgcagtcgacggtaccgcgggccc  gccaccatgtctgaatatattcg-3’ | | 5’-tctagagtcgcgggatcccctacattcc  ccagccagaagac-3’ |
| **NUP188** | Cryptic exon amplification | | 5’-ggagcagtagagaactgtgg-3’ | | 5’-gctgattcttaaacccagttcc-3’ |
| **POLDIP3** | Alternatively spliced exon amplification | | 5’-gcttaatgccagaccgggagttg-3’ | | 5’-tcatcttcatccaggtcatataaatt-3’ |
