# Supplementary material for: Pleiotropic requirements for human TDP-43 in the regulation of cell and organelle homeostasis
Source: Life Sci Alliance. 2019 Sep 16;2(5):e201900358. doi: 10.26508/lsa.201900358 (PMC6749094; doi:10.26508/lsa.201900358)
Supplement: Supplementary file 4 [file LSA-2019-00358_TableS4.doc]

**Supplemental Table 4: Summary of antibodies used in this study.**

| **Antibody** | **Source** | **Catalog/Clone number** |
| --- | --- | --- |
| **Cathepsin D** | R&D Systems | AF1014 |
| **Cathepsin L** | R&D Systems | AF 952 |
| **Cytochrome C** | BD Pharmingen | 556432 |
| **EEA1** | Thermo Fischer Scientific | PA1-063A |
| **GM130** | BD Transduction Laboratories | 610822 |
| **LAMP1** | University of Iowa DSHB | H4A3 |
| **Nucleoporin p62** | BD Biosciences | 610497 |
| **Nup188** | Bethyl Laboratories | A302-322A |
| **PFKP** | Cell Signaling | 5412S |
| **PGRN** | R&D Systems | AF2420 |
| **POLDIP3 (SKAR)** | Proteintech | 17466-1-AP |
| **Saposin C** | Santa Cruz Biotechnology | sc-32875 |
| **Ribosomal Protein S6** | Cell Signaling Technology | 2217 |
| **TDP43** | Cell Signaling Technology | 3449S |
| **TIAR** | Cell Signaling Technology | 5137S |
| **Tubulin** | Sigma -Aldrich | T5168 |
